# Nuclear introgression without mitochondrial introgression in two turtle species exhibiting sex‐specific trophic differentiation

**DOI:** 10.1002/ece3.2087

**Published:** 2016-04-12

**Authors:** Sarah M. Mitchell, Laura K. Muehlbauer, Steven Freedberg

**Affiliations:** ^1^ Department of Ecology, Evolution, and Organismal BiologyIowa State UniversityAmesIowa50011‐1020; ^2^ Department of BiologySt. Olaf College1520 St. Olaf AvenueNorthfieldMinnesota55057

**Keywords:** *Graptemys*, hybridization, introgression, stable isotope analysis, turtles

## Abstract

Despite the presence of reproductive barriers between species, interspecific gene introgression has been documented in a range of natural systems. Comparing patterns of genetic introgression in biparental versus matrilineal markers can potentially reveal sex‐specific barriers to interspecific gene flow. Hybridization has been documented in the freshwater turtles *Graptemys geographica* and *G. pseudogeographica*, whose ranges are largely sympatric. Morphological differentiation between the species is restricted to females, with female *G. geographica* possessing large heads and jaws compared to the narrow heads of *G. pseudogeographica* females. If hybrid females are morphologically intermediate, they may be less successful at exploiting parental feeding niches, thereby limiting the introgression of maternally inherited, but not biparental, molecular markers. We paired sequence data with stable isotope analysis and examined sex‐specific genetic introgression and trophic differentiation in sympatric populations of *G. geographica* and *G. pseudogeographica*. We observed introgression from *G. pseudogeographica* into *G. geographica* at three nuclear loci, but not at the mitochondrial locus. Analysis of ∂^15^N and ∂^13^C was consistent with species differences in trophic positioning in females, but not males. These results suggest that ecological divergence in females may reduce the opportunity for gene flow in this system.

## Introduction

When leaks in reproductive isolation occur in nature, studying hybridizing species can shed light on how barriers between species are formed and maintained (Good et al. 2008; Cristescu et al. 2010; De Leon et al. 2010; Mitsui et al. 2011). While hybrid fitness can be studied by directly examining the survival or fecundity of F1 hybrids (Schluter 2000), quantifying patterns of genetic introgression between sympatric species using molecular markers can offer insight into the long‐term movement of genes across species barriers. For instance, limited introgression in hybrid zones can point to selection against intermediate hybrid phenotypes (Rawson et al. 1999; Brannock et al. 2009). Comparing rates of introgression for biparentally versus uniparentally inherited markers offers the opportunity to further investigate the forces acting to maintain reproductive isolation. Sex differences in hybrid fitness have been implicated in the differential introgression of nuclear and mitochondrial (mt) markers in animal populations (Good et al. 2008; Zalapa et al. 2009; Snow et al. 2010). A general trend toward elevated rates of introgression of mt and chloroplast DNA relative to nuclear DNA has been documented across plant and animal taxa, a pattern attributed to reduced reproductive success of hybrid males (Chan and Levin 2005).

A number of behavioral and genetic factors can contribute to reproductive barriers between species in nature, yet the effect of ecological forces on reproductive isolation is less clear. If parental species occupy distinct ecological niches and exhibit different morphologies, hybrid offspring can have a morphology intermediate to that of their parents, making them less competitive at exploiting either parental niche and inhibiting gene flow between the species (Benkman 1993; Schluter 1993, 1995, 2001; Craig et al. 1997). Reduced hybrid fitness in hybrid zones can result in reproductive isolation between recently diverged lineages, theoretically contributing to speciation by maintaining genetic differentiation of the lineages (Wilson and Turelli 1986; Rice and Hostert 1993; Rundle and Whitlock 2001). Alternatively, gene flow may still occur despite reduced fitness of early generation hybrids, potentially allowing for the establishment of novel evolutionary lineages or an increase in the adaptive potential of parental species (Rieseberg 1997; Arnold et al. 1999; Grant and Grant 2008). The ability of hybrids to compete in parental environments is greatly impacted by the degree of ecological niche differentiation of the parental species (Nosil and Harmon 2008).

An animal's ecological niche encompasses a broad range of biotic and abiotic factors, and the feeding niche has a particularly important impact on a species' role in the ecosystem (Syväranta and Jones 2008; Crow et al. 2010). Feeding niches have historically been studied through gut and fecal content; however, these methods capture a temporally limited perspective into the animal's diet and are biased by variable digestion rates of consumed food sources (Inger and Bearhop 2008; Toscano et al. 2010). For example, diets of some turtles of the genus *Graptemys* are comprised partly of mollusks, and diet inferred from gut content analysis may be heavily biased by the indigestibility of mollusk shells (Bulté et al. 2008a). In contrast, stable isotope analysis can provide long‐term insight into dietary sources and trophic positioning, particularly for tissues with low turnover rate (Crawford et al. 2008; Inger and Bearhop 2008; Pinela et al. 2010). The lighter isotope of nitrogen (^14^N) is preferentially excreted relative to the heavy isotope (^15^N), and thus, the ratio of these isotopes (denoted ∂^15^N) in animal tissue can be used as an indicator of trophic level (Crawford et al. 2008; Inger and Bearhop 2008). Ratios of nitrogen isotopes in animal tissue can be compared with those of primary consumers to quantify trophic positioning (Syväranta and Jones 2008; Doi et al. 2010; Pinela et al. 2010). Many studies couple ∂^15^N analysis with analysis of ratios of stable isotopes of carbon (^13^C:^12^C, denoted ∂^13^C), allowing for distinctions among carbon sources in animal diets. Overlap in both ∂^15^N and ∂^13^C signatures can be used to infer dietary overlap among sympatric species (Hodum and Hobson 2000).

Despite having diverged 5–8 million years ago, the emydid turtles *Graptemys geographica* and *G. pseudogeographica* are capable of hybridization in the wild (Vogt 1978 cited in Fritz 1995; Freedberg and Myers 2012). Previous findings suggest that males and females do not discriminate between species in mate‐choice experiments (Myers 2008) and hybrid offspring between these species are viable and fertile (Freedberg and Myers 2012). Haplotypes at one mt and two nuclear loci distinguishing *G. geographica* from *G. pseudogeographica* across their ranges were found to have introgressed in an isolated population of *G. pseudogeographica* and coalescent simulations allowed incomplete lineage sorting to be rejected as a cause for the shared haplotypes (Freedberg and Myers 2012). *Graptemys geographica* females exhibit enlarged heads and strong jaws that have been proposed as adaptions to a molluscivorous diet (Lindeman 2006; Bulté et al. 2008a), while *G. pseudogeographica* females in northern populations have significantly narrower heads, likely adapted to consume plants and soft‐bodied arthropods (Vogt 1981; Lindeman 2000). Males of the species have similar head morphology and body size and appear to share a common dietary niche (Vogt 1981).

In this study, we examined sex‐specific introgression by sequencing one mt and three nuclear markers in sympatric populations of *G. pseudogeographica* and *G*. *geographica*. To understand the selective forces that may be impacting the fate of hybrid turtles, we used stable isotope analysis to quantify trophic positioning and dietary carbon sources for both species and sexes. If morphological differences between females of the two species are associated with strong dietary divergence, hybrid females may exhibit an intermediate phenotype that places them at a competitive disadvantage relative to pure parental females. Although these morphological differences may be difficult to directly observe whether hybridization is rare in the wild, reduced fitness of female hybrids relative to male hybrids is expected to be associated with reduced mt introgression relative to nuclear introgression.

## Methods

During the summers of 2007–2012; 140 adult map turtles (76 *G. geographica* and 64 *G. pseudogeographica*) were collected by hoop trapping and hand collection from the Mississippi River near Kellogg, MN, Wabasha co. The collecting site contains large sympatric populations of both species with some microgeographical overlap; individual traps frequently captured turtles of both species and sexes. These species of *Graptemys* turtles are easily distinguished by head patterning, eye and skin pigmentation, and shell morphology (reviewed in Ernst et al. 1994). Several *Graptemys ouachitensis* were captured but not included in this study. Each turtle was classified by species, sex, and age and was uniquely marked for future identification prior to release. Tissue samples were taken from the tail tip and preserved in 95% ethanol at −20°C prior to stable isotope analysis and DNA extraction.

### Genetic analysis

Species‐specific genetic markers have previously been developed at one mt and two nuclear loci that are capable of distinguishing between *G. geographica* and *G. pseudogeographica* (Freedberg and Myers 2012). We identified one additional nuclear locus for an intron for a transcription growth factor (TGF) that exhibited strong species‐specificity in samples downloaded from GenBank. We developed primers for the TGF locus and tested the markers for species‐specificity in additional representative samples across the two species' ranges. TGF primers were tested on a total of 15 *G. geographica* samples and 12 G. *pseudogeographic/ouachitensis* samples. Previous analyses at other loci have failed to reveal any species‐specific genetic divergence between *G. pseudogeographica* and *G. ouachitensis* (Freedberg and Myers 2012).

DNA was extracted with a Puregene DNA extraction kit for cells and tissue (Gentra Corporation, Minneapolis, MN). Three nuclear loci were studied: ODC4 (5′‐GGGTTTCTTTCAATTGCTGTAGTAA‐3′ and 5′‐CAGAGCACCGCTGGGAAT‐3′) amplified a 464‐bp fragment of an intron of the ornithine decarboxylase antizyme, HNFAL (5′‐CAGCAATGATAGAACCCAGGA‐3′ and 5′‐GATGACAGCCACATTCGTTC‐3′) amplified a 220‐bp fragment of an intron from the hepatocyte nuclear factor, and TGF (5′‐CCACCAGTACTAGTCCCCAGTC‐3′ and 5′‐GCTGTAATTCTTTAAACCATGAGCTA‐3′) amplified a 290‐bp fragment of an intron from a transforming growth factor. A 387‐bp fragment of the mt control region was amplified using primers developed by M. Sorenson at the University of Massachusetts (5′‐CAAGGGTGGATCGGGCATAAC‐3′ and 5′‐GTGCCTGAAAAAACAACCACAGG‐3′, Freedberg et al. 2005). All four markers were run on samples from *G. geographica*. After detecting negligible introgression at the HNFAL, ODC, and mt loci, TGF was not tested in *G. pseudogeographica*.

Polymerase chain reaction (PCR) was performed in a 10 μl reaction consisting of approximately 0.5 μl of 30 μmol/l DNA, 1 μl 10X tricine *Taq* buffer, 1 μmol/l MgCl_2_, 0.005 μg of each primer, 100 mmol/l of each dNTP, and 0.1 unit *Taq* polymerase. An initial denaturation (8 min at 95°C) was followed by 35 cycles of 45 sec at 95°C, 1 min at the annealing temperature (mt: 55°C; HNFAL: 53°C, ODC: 58°C, TGF: 53°C), 1 min at 72°C, followed by a final 8 min extension at 72°C. Amplified PCR products were sent to either the University of Washington High Throughput Sequencing Facility or the Plant–Microbe Genomics Facility at Ohio State University in Columbus, Ohio to be sequenced. PCR products were purified with EXO‐SAP enzymatic incubation or with the AMPure reagent (Beckman Coulter, Brea, CA). Sequencing was performed using the ABI BigDye v3.1 ([2 min at 96°C, 15 sec at 50°C, and 4 min at 60°C, followed by 25 cycles of 30 sec at 96°C, 15 sec at 50°C, and 4 min at 60°C] or [1 min at 96°C, then 45 cycles of: 10 sec at 96°C, 5 sec at 50°C and 4 min at 60°C]). Sequencing cleanup was performed with ABI BigDye Xterminator kit or HighPrep DTR magnetic beads (MagBio Genomics, Gaithersburg, MD). Sequence analysis was performed on an ABI 3730 or ABI3730xl DNA Analyzer.

Sequences were processed, aligned, and analyzed with Geneious 4.5.5. All sequences aligned perfectly with alleles previously found to characterize either *G. geographica* or *G. pseudogeographica* (Freedberg and Myers 2012). Haplotype frequencies were calculated by dividing the number of copies of each haplotype by the total number of copies of each genome (*n* for mt DNA, 2*n* for nuclear DNA). A Fisher's exact test was run to test for differences in the frequencies of introgressed haplotypes at each locus, as well as differences in haplotype frequencies at the same locus between the species. Tests for linkage disequilibrium and departures from Hardy–Weinberg equilibrium were run in Arlequin 3.5 (Excoffier and Lischer 2010).

### Stable isotope analysis

Tissue samples from a subset of each sex and species were used in stable isotope analysis. Each sample was sliced into several thin pieces and dried at 50°C for at least 48 h, after which a 0.2–0.7 mg sample was weighed and processed. Samples were run on an isotope‐ratio mass spectrometer (Elemental Combustion System, Costech Instruments, Model 4010 linked to a ThermoScientific, Rockford, IL, USA, Delta V Mass Spectrometer) to test for isotopic nitrogen (∂^15^N) and carbon (∂^13^C) levels. Stable isotope analysis was run on 11 *G. geographica* females and 16 *G. pseudogeographica* females, and 24 *G. geographica* males and 35 *G. pseudogeographica* males. ∂^15^N was similarly analyzed for two plant specimens (*Lemna* sp.) from the study site to quantify trophic position for turtles using the formula:

$$\text{TP}=1+\left(\frac{\partial^{15}N\;turtle-\partial^{15}N\;Lemna}{\Delta \partial^{15}N}\right)$$

with a discrimination factor (Δ∂^15^N) of 3.4 (Post 2002; Martinez del Rio et al. 2009). For each sex, pairwise *t*‐tests were run to test for differences in ∂^15^N, ∂^13^C and trophic position between *G. geographica* and *G. pseudogeographica*.

## Results

### Genetic analysis

Most genetic sequences yielded clear, unambiguous reads across all informative nucleotide positions. Samples that failed to amplify or were unclear were not used. A subset of samples was sequenced multiple times to confirm haplotypes. The TGF marker exhibited clear species‐specificity in the representative samples of each species: all 12 *pseudogeographica/ouachitensis* were homozygous for guanine at position 63, while 14 of 15 *G. geographica* were homozygous for adenine at this position. One *G. geographica* from an area of range overlap between *G. geographica* and *G. pseudogeographica* was heterozygous for the two alleles and likely represents a case of hybridization between sympatric populations of the species (Appendix).

All of the Minnesota sequences aligned unambiguously with one of the species‐specific haplotypes identified in Freedberg and Myers (2012) or the haplotypes derived from representative samples for the TGF locus. For *G. geographica*, 75 samples produced clear reads at the mt locus, 73 samples produced clear reads at the HNFAL locus, 70 samples produced clear reads at the ODC locus, and 70 samples produced clear reads at the TGF locus. For *G. pseudogeographica*, 63 samples produced clear reads at the mt locus, 59 samples produced clear reads at the HNFAL locus, and 62 samples produced clear reads at the ODC locus. Most specimens displayed haplotypes consistent with their phenotype at all four loci. Hardy–Weinberg tests showed no departure from HW equilibrium for any locus in either species (*P* > 0.05 for all loci). No linkage disequilibrium was detected between any pairs of loci (*P* > 0.05 for all comparisons).

In *G. geographica*, introgression was detected at all three nuclear loci; the haplotype frequency of the *G. pseudogeographica* haplotype into *G. geographica* was 0.079 at the ODC locus, 0.034 at the HNFAL locus, and 0.21 at the TGF locus (Table 1). In *G. pseudogeographica*, only one individual was heterozygous at the ODC locus (haplotype freq. = 0.008) and no introgression was detected at the HNFAL locus. No mt introgression was detected in either species. The frequency of *G. pseudogeographica* haplotypes into *G. geographica* was significantly greater at the ODC locus than the *mt* locus (*P* = 0.009) and at the TGF locus than the *mt* locus (*P* < 0.0001). There was no difference in haplotype frequencies between HNFAL and mt loci (*P* = 0.16) or between HNFAL and ODC (*P* = 0.126). Haplotype frequency of the introgressed allele at the TGF locus was greater than at the HNFAL locus (*P* < 0.0001) and the ODC locus (*P* = 0.002).

**Table 1:** Frequency of species‐specific nuclear and mitochondrial genotypes found in *Graptemys geographica* and *G. pseudogeographica*

*G. geographica*		
ODC (nuclear)		
Genotype	Frequency	%
*Gg*/*Gg*	59	84.3
*Gg*/*Gp*	11	15.7
*Gp*/*Gp*	0	0

HNFAL (nuclear)		
Genotype	Frequency	%
*Gg/Gg*	69	93.2
*Gg/Gp*	3	4.1
*Gp/Gp*	1	1.4

TGF (nuclear)		
Genotype	Frequency	%
*Gg/Gg*	43	61.4
*Gg/Gp*	24	34.3
*Gp/Gp*	3	4.3

mt		
Haplotype	Frequency	%
*Gg*	75	100
*Gp*	0	0

*G. pseudogeographica*		
ODC (nuclear)		
Genotype	Frequency	%
*Gp/Gp*	61	98.4
*Gp/Gg*	1	1.6
*Gg/Gg*	0	0

HNFAL (nuclear)		
Genotype	Frequency	%
*Gp/Gp*	59	100
*Gp/Gg*	0	0
*Gg/Gg*	0	0

mt		
Haplotype	Frequency	%
*Gp*	63	100
*Gg*	0	0

The haplotype frequency of the introgressed ODC allele was significantly greater from *G. pseudogeographica* into *G. geographica* than vice versa (*P* = 0.006). The *G. pseudogeographica* HNFAL haplotype was also more common in *G. geographica*, than vice versa, although this difference was not statistically significant (*P* = 0.067).

### Stable isotope analysis

**Figure 1:**
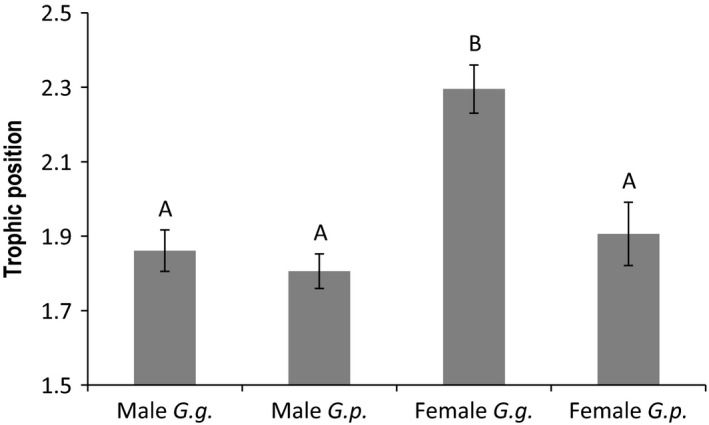
Trophic position (±1 SE) was significantly elevated in female *Graptemys geographica* compared to female *G. pseudogeographica*,* male G. pseudogeographica*, and male *G. geographica*. No species difference was observed in males. Different letters above bars denote statistically significant differences (*P* < 0.05).

Mean ∂^15^N and trophic position of female *G. geographica* was significantly higher than that of female *G. pseudogeographica* (df = 25; *t* = 3.36; *P* = 0.002 for both analyses). Despite the substantially larger number of samples analyzed, no species difference was detected in ∂^15^N or trophic position for male turtles (Fig. 1; df = 57; *t* = 0.76; *P* = 0.45 for both analyses). No differences were detected between *G. pseudogeographica* females and either *G. pseudogeographica* or *G. geographica* males (*P* > 0.69 for both analyses). There was no difference in ∂^13^C between *G. geographica* and *G. pseudogeographica* for either sex (male: df = 57; *t* = 0.51; *P* = 0.61, female: df = 25; *t* = −1.2; *P* = 0.24).

## Discussion

### Trophic differentiation and “competitive valleys” for hybrids

Stable isotope analysis revealed a clear sex‐specific pattern of trophic differentiation. Female *G. geographica* were characterized by significantly higher ∂^15^N values relative to female *G. pseudogeographica*, reflecting higher trophic positioning in *G. geographica* (Fry 2006). The similarity in ∂^13^C values indicates diets derived from a common carbon source, and taken in concert with the ∂^15^N data, points to species feeding at differing trophic levels in the same primary producer community (Hofmeister and Freedberg 2013). If larger head widths in female *G. geographica* are adapted for feeding primarily on omnivorous molluscs, the lower trophic positioning of *G. pseudogeographica* females reflects a diet comprised of more herbivorous prey and/or plant material. Although earlier researchers have speculated that the variation in head size between related species is a plastic response to a molluscivorous diet (Vogt 1981; Fachin‐Teran et al. 1995), the presence of consistent head width differences among hatchlings of different *Graptemys* species strongly points to a genetic control of this phenomenon (Lindeman 2000).

In contrast to females, males exhibited no species differences in ∂^15^N and were characterized by ∂^15^N values similar to female *G. pseudogeographica* (Fig. 1). In concert with similar ∂^13^C profiles, these results suggest that males of the two species are feeding on similar resources. Although male *G. geographica* exhibit broad head widths in some parts of their range (Lindeman 2000), males of both species at our study site have similarly narrow head widths (personal observation) and occupy a similar trophic positioning, comparable to that of *G. pseudogeographica* females. Stable isotope analysis of an isolated lake population of *G. geographica* showed considerable overlap in diet between adult males and females (Bulté et al. 2008b), although it is possible that interspecific competition with *G. pseudogeographica* may drive dietary specialization of *G. geographica* females in other populations (Lindeman 2000).

When hybrid offspring coexist with both parental species, competitive interactions can select against morphologically intermediate hybrids, particularly in the absence of alternate habitats for hybrids to exploit (reviewed in Rundle and Nosil 2005). Because F1 hybrids exist for only a single generation, observing hybrid individuals in populations with low introgression frequencies may be unlikely (Guadagnuolo et al. 2001). In other turtle species, confirmed interspecific F1 hybrids consistently exhibit phenotypes intermediate of the parental species, including head morphology (Galgon and Fritz 2002; Seminoff et al. 2003; Xia et al. 2011). The significant morphological and trophic divergence of female *G. geographica* and *G. pseudogeographica* in this system suggests that hybrid females may be less capable of exploiting either parental niche relative to pure parental females. Schluter (1993) found that hybrids of benthic and limnetic sticklebacks possessed an intermediate morphology, placing them at competitive disadvantage relative to either parental species in their respective feeding niches. Hybrids sticklebacks suffer significantly reduced survival in the field relative to parentals (Gow et al. 2007). A similar pattern has been observed in pea aphids, where F1 hybrids have reduced fitness relative to parentals when reared on either parental food source (Via et al. 2000). The lack of trophic and morphological differentiation between *G. geographica* and *G. pseudogeographica* males suggests that hybrid male offspring are not prevented from utilizing either parental food source. Although the trophic and molecular introgression patterns that we observed are consistent with reduced survival of female hybrids, data detailing morphological differences between pure and hybrid specimens would lend valuable additional support to this scenario. Future studies that encourage interspecific matings in the laboratory (Fritz 1995) may allow morphology and competitive abilities of hybrid offspring to be quantified in this system.

### Patterns of introgression

Nucleotide sequencing indicates that hybridization events have occurred between sympatric populations of *G. geographica* and *G. pseudogeographica* at our study site. Linkage equilibrium among the *G. pseudogeographica* nuclear markers in *G. geographica* suggests that hybrid offspring are fertile and have backcrossed with “pure” *G. geographica* for several generations. While previous research has reported interspecific introgression at these loci in an allopatric population of *G. pseudogeographica kohnii* (Freedberg and Myers 2012), the present study documents introgression in a population characterized by substantial habitat overlap between these species.

Introgression was observed from *G. pseudogeographica* into *G. geographica* at all three nuclear loci examined, while mt introgression was completely absent. The contrasting pattern of introgression in the mt versus nuclear loci is consistent with selection against intermediate hybrid phenotypes in female, but not male turtles. The divergent trophic specialization of female *Graptemys* may limit the ability of F1 hybrid females to compete with either parental species in their respective feeding niche, inhibiting the opportunity for mt introgression. In Tennessee, where introgression has occurred between these species in the absence of competition with pure *G. geographica*, there was no difference in the rate of mt versus nuclear introgression from *G. geographica* into *G. pseudogeographica* (Freedberg and Myers 2012).

Although the pattern of introgression observed is consistent with extrinsic selection against intermediate female hybrids, it is important to entertain alternate phenomena that may result in nuclear introgression in the absence of mt introgression. Genetic interactions involving the sex chromosomes (Haldane's Rule) can reduce the fitness of the heterogametic sex, producing mito‐nuclear introgression disparity (Cianchi et al. 2003; Carling and Brumfield 2008). Turtles of the genus *Graptemys* have temperature‐dependent sex determination (Bull and Vogt 1979) and thus are not impacted by sex chromosomal barriers to gene flow. Cytonuclear discordance may also result in intrinsic selection against introgressed mitochondria, inhibiting the movement of mt DNA between species (Burton et al. 2006). The finding of no difference in the levels of mt and nuclear introgression between *G. geographica* and *G. pseudogeographica* in a separate population suggests that cytonuclear disruption is not inherently deleterious in this system (Freedberg and Myers 2012). Positive selection on linked nuclear alleles could theoretically favor elevated introgression at nuclear loci, although the absence of linkage disequilibrium indicates that our markers are not physically linked, and thus, multiple independent instances of positive selection would be required to explain our results. Greater nuclear than mt introgression may also occur if males discriminate against female hybrids; however, mate‐choice studies reveal that *G. geographica* males do not discriminate between *G. pseudogeographica* and *G. geographica* females (Myers 2008).

Introgression between the species was strongly asymmetrical; we found only one instance of introgression from *G. geographica* into *G. pseudogeographica* at the ODC locus and none at the HNFAL or mt loci, indicating that male hybrids are primarily backcrossing into the *G. geographica* population. Preferential backcrossing into *G. geographica* cannot be explained by the relative abundances of the parental species, as *G. pseudogeographica* is more common than *G. geographica* at our study site (Pappas et al. 2001). Unidirectional introgression is often attributed to habitat preferences of hybrids (Hapke et al. 2011), or species differences in mate choice (Hartog et al. 2010; Peterson et al. 2011). While *G. geographica* into *G. pseudogeographica* show considerable habitat overlap at our site, *G. pseudogeographica* have a keeled carapace and often venture into the main channel of the river, while *G. geographica* have an unkeeled shell and are generally confined to calm backwater areas. Keels can provide stability for aquatic animals in turbulent water (Bartol et al. 2003), and thus if hybrids exhibit reduced keels, they may be restricted to the slower current backwater, facilitating introgression into *G. geographica*. Differences in mating behavior may further facilitate unidirectional introgression. *G. pseudogeographica* males touch the females' heads with their forelimbs to entice the females into mating, a behavior that is absent in *G. geographica* (Vogt 1980). Failure of hybrids to exhibit this behavior may result in *G. pseudogeographica* females being less receptive to hybrid males than *G. geographica* females.

Interspecific introgression can have a range of potential impacts on populations of hybridizing species. Hybrids may displace parental genotypes if their fitness exceeds that of both pure parentals or they may become established alongside parentals in a niche that favors hybrids over either parental phenotype (Rieseberg 1997). In addition, the habitat disruption often associated with hybridization can increase the opportunity for hybrid lineages to adaptively radiate (Seehausen 2004). More commonly, early generation (e.g., F1, F2, and B1) hybrids suffer reduced fitness relative to parentals owing to the disruption of coadapted gene complexes. This pattern can result in narrow hybrid zones in areas of introgression, with parental species' identities remaining intact (Rieseberg 1997). It is generally underappreciated that even in the face of reduced fitness of early generation hybrids, extensive gene flow between hybridizing species may still occur, owing to genetic drift or positive selection on certain genetic elements (Arnold et al. 1999). Despite periods of selection against F1 hybrids, interspecific gene flow can increase the adaptive potential of introgressing species via the introduction of novel genetic variation (Grant and Grant 2008).

## Conclusions

Despite existing sympatrically over most of their ranges, *G. pseudogeographica* and *G. geographica* are capable of hybridizing and introgressing genes across species barriers. When hybrid offspring among sympatric populations are fertile and viable, hybridization can result in the assimilation of the hybridizing species or extinction of one parental species via demographic swamping (Wolf et al. 2001). Despite these predictions, there are numerous examples of species identities apparently being maintained despite ongoing introgression (De Busschere et al. 2010; De Leon et al. 2010; Mitsui et al. 2011). Habitat specialization has been found to maintain partial reproductive isolation among populations in the face of limited gene flow (Via 1999; De Busschere et al. 2010), and assortative mating may increase reproductive isolation among sympatric populations (Caillaud and Via 2000; Pennings et al. 2008; De Leon et al. 2010; Pfennig and Pfennig 2010). *G. pseudogeographica* and *G. geographica* have remained distinct despite years of sympatry and incomplete genetic and behavioral barriers to gene flow. While our results suggest that interspecific gene flow can occur between these sympatric species, the strong dietary specialization of females may be associated with a reduction in gene flow, helping to maintain species boundaries. Examining patterns of trophic differentiation between these species in ranges of sympatry versus allopatry may ultimately shed light on the forces contributing to species distinctness in this system.

## Conflict of Interest

None declared.

## Appendix

**Table:**

ID	Species	Genotype	Collecting location
272	G	AA	Ohio, USA
59643	G	AA	Alabama, USA
27818	G	AA	Kentucky, USA
23397^a^	G	AA	Indiana, USA
59527	G	AA	Illinois, USA
59646	G	AA	Illinois, USA
60056	G	AA	Iowa, USA
60057	G	AA	Iowa, USA
59524	G	AA	Missouri, USA
59657	G	AA	Missouri, USA
59658	G	AA	Missouri, USA
59666	G	AA	Missouri, USA
59667	G	AA	Missouri, USA
59668	G	AA	Missouri, USA
60001	G	AG	Missouri, USA
23217	P	GG	Tennessee, USA
23218	P	GG	Tennessee, USA
59639	P	GG	Tennessee, USA
59999	P	GG	Illinois, USA
11150^a^	P	GG	No locality data
646	P	GG	Minnesota, USA
Gp6	P	GG	Minnesota, USA
LK	O	GG	Indiana, USA
23347^a^	O	GG	Tennessee, USA
59635	O	GG	Illinois, USA
52064	O	GG	Wisconsin, USA
35214	O	GG	Arkansas, USA

a Denotes sequence downloaded from GenBank.
